# Distribution and species composition of zooplankton (rotifers and crustaceans) in the Basin of the Middle Volga River, Russia

**DOI:** 10.3897/BDJ.9.e76455

**Published:** 2021-11-09

**Authors:** Oksana Mukhortova, Stepan Senator, Elena Unkovskaya

**Affiliations:** 1 Samara Federal Research Center of the Russian Academy of Sciences, Institute of the Ecology of the Volga River basin of the Russian Academy of Sciences, Togliatti, Russia Samara Federal Research Center of the Russian Academy of Sciences, Institute of the Ecology of the Volga River basin of the Russian Academy of Sciences Togliatti Russia; 2 Tsitsin Main Botanical Garden of the Russian Academy of Sciences, Moscow, Russia Tsitsin Main Botanical Garden of the Russian Academy of Sciences Moscow Russia; 3 Volzhsko-Кamsky State Nature Biosphere Reserve, Sadoviy, Russia Volzhsko-Кamsky State Nature Biosphere Reserve Sadoviy Russia

**Keywords:** dataset, occurrence, zooplankton, Rotifera, Crustacea, field studies, abundance, Middle Volga River

## Abstract

**Background:**

The presented dataset contains information on the distribution and species composition of zooplankton (rotifers and crustaceans) registered in the Basin of the Middle Volga River, Russia. The studies have been performed in the Kuibyshev Reservoir (Samara Oblast and the Republic of Tatarstan), the Saratov Reservoir (Samara Oblast), in several lakes (Raifskoe, Gniloe, Krugloe and Lenevo) in the Volzhsko-Kamsky State Biosphere Reserve (Republic of Tatarstan) and in Lake Aslikul, one of the largest lakes of the Middle Volga River Basin, located in the Asly-Kul Natural Park (Republic of Bashkortostan). The hydrobiological data were obtained and published from 1957 to 2020. In total, the dataset includes 5141 records of 111 zooplankton species (including 17 subspecies), belonging to 45 genera. These are mainly native species - 98.5%, while the naturalised, including invasive species, accounts for less than 1.5%.

**New information:**

A total of 5141 records have been published on the taxonomic diversity and occurrence of zooplankton (rotifers and crustaceans) in the Middle Volga River Basin. Each record includes information about the place and date of finding the specimen, its taxonomy, occurrence and abundance and the collector. If the information about the find has been published, a link to the corresponding reference is provided. The presented dataset supplements the data on the distribution of zooplankton species in the European part of Russia. Data on zooplankton in the Middle Volga River Basin are published for the first time.

## Introduction

Zooplankton is the main consumer of microalgae, which plays an important role in the transformation of organic matter (Gerasimova et al. 2021, Ivanova and Kazantseva 2006). Planktonic animals mineralise organic matter and release metabolites to the external environment, which are further utilised by bacteria and algae. Zooplankton representatives are one of the few aquatic organisms that utilise microalgae, bacteria and detritus and then transfer this energy to higher trophic levels. They are food objects for larger invertebrates and fish (Gerasimova et al. 2021, Shurganova et al. 2021). This publication presents a prepared dataset on the current diversity and distribution of zooplankton species in the reservoirs of the Middle Volga River (European Russia). The dataset contains information on the locations and abundance of approximately 38% of species known for the Middle Volga River Basin (e.g. Timokhina 2000, Popov and Mukhortova 2016, Lazareva 2020, Shurganova et al. 2021). The dataset records refer mainly to native species (98.5%); naturalised species, including invasive species, account for no more than 1.5% (Mukhortova et al. 2021).

## General description

### Purpose

The study aims to present a published dataset containing information on the zooplankton distribution, species composition and abundance (rotifers and crustaceans) in the Middle Volga River Basin (European part of Russia).

## Project description

### Title

Distribution and species composition of zooplankton (rotifers and crustaceans) in the Basin of the Middle Volga River, Russia

### Personnel

Oksana Mukhortova, Stepan Senator, Elena Unkovskaya

### Study area description

**Figure 1.**
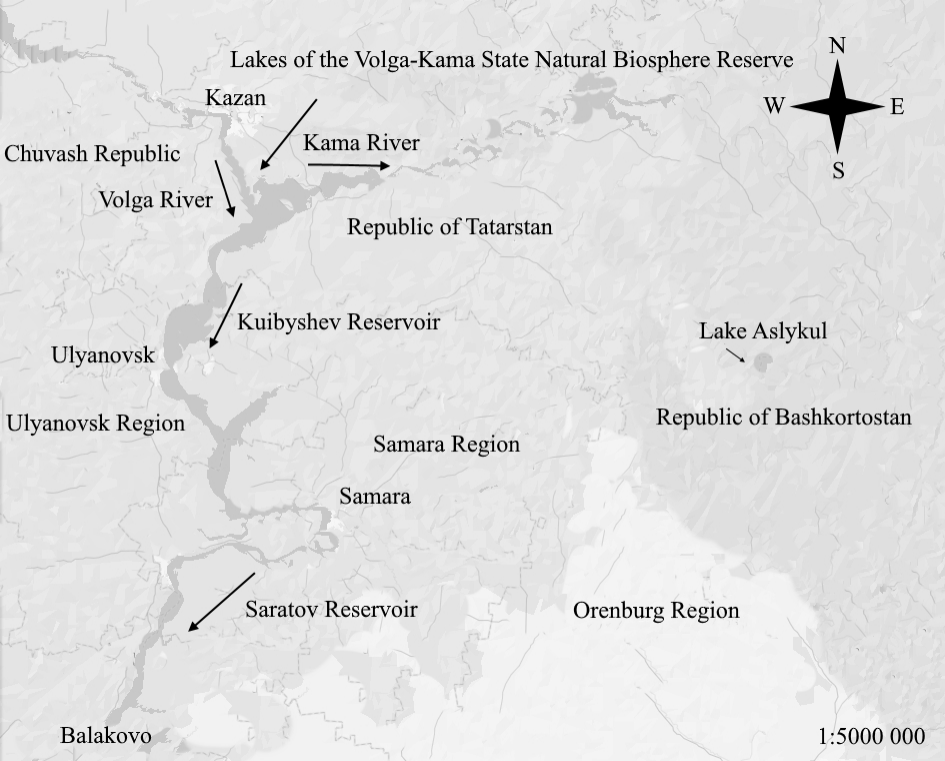
Layout of the investigated water bodies.

The studies covered the Kuibyshev Reservoir (Samara Oblast and the Republic of Tatarstan), the Saratov Reservoir (Samara Oblast), several lakes (Raifskoe, Gniloe, Krugloe and Lenevo) in the Volzhsko-Kamsky State Biosphere Reserve (Republic of Tatarstan) and Lake Aslikul, one of the largest lakes of the Middle Volga River Basin, located in the Asly-Kul Natural Park (Republic of Bashkortostan) (Fig. 1).

1. Kuibyshev Reservoir, located on the Volga River, is the largest water reservoir in Eurasia. It was established and launched in 1955—1957, shortly after the construction of the dam of the Volzhskaya hydroelectric power station, named after V.I. Lenin (currently, Zhigulevskaya HPP), had been finished. Saratov Reservoir is a large reservoir on the Volga River, formed by the dam of the Saratov HPP and it was launched in 1967—1968. The Kuibyshev and Saratov Reservoirs are similar by their origin and are artificial reservoirs. The bottom is mainly muddy, some areas are sandy or stony, there are some areas with snag accumulations. The water in the reservoirs is fresh, hydrocarbonate-calcium, average salinity 0.3–0.6 g/l, water transparency 1.0–1.6 m, average colour is 50–60 degrees of the platinum-cobalt scale.
2. The water of all the lakes of the Volzhsko-Kamsky Biosphere Reserve belongs to the hydrocarbonate class of the calcium group with low and medium degree of mineralisation. The waters are characterised as “soft” and “moderately hard” (WQA). The environmental pH varies between the lakes, depths and seasons from slightly acidic (6.5–6.9) to slightly alkaline (7.5–8.3), reaching maximum values (8.8–10.0) during the period of phytoplankton and cyanobacteria blooms. The gas regime of the lakes in summer is typical for eutrophic water bodies: in the surface layers, the dissolved oxygen saturation varies as 72–226.9% and in the bottom layers, there is an oxygen deficiency (7–46%).
3. The water in Lake Aslykul is slightly brackish with high salinity (1.94 g/l). The Sharlama Stream flows into the Lake. When the water level is high, the Asily-Udryak Stream flows out of the Lake.

**Figure 2.**
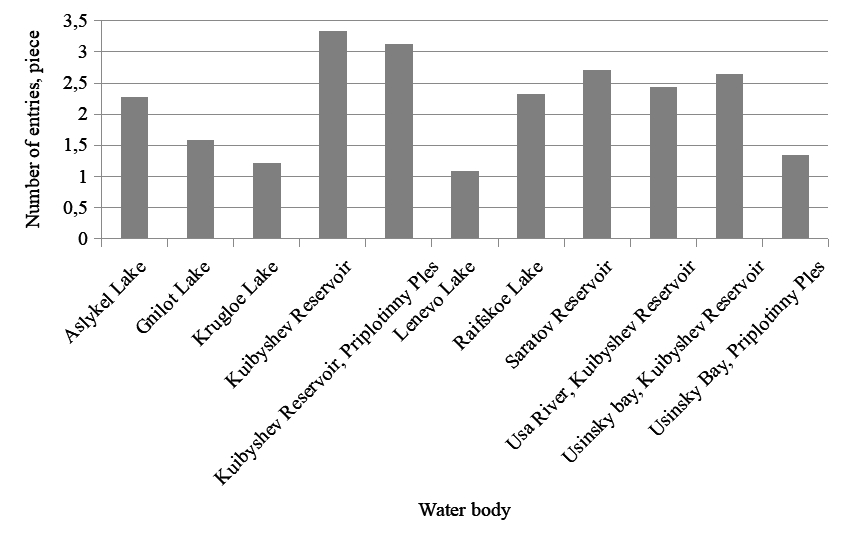
The number of records for each of the studied water bodies.

The distribution of the number of records on the species composition and abundance of zooplankton for the Kuibyshev Reservoir (81% in total), the Saratov Reservoir (10%) and in the Lakes (5%) is presented below (Fig. 2).

### Design description

**Figure 3.**
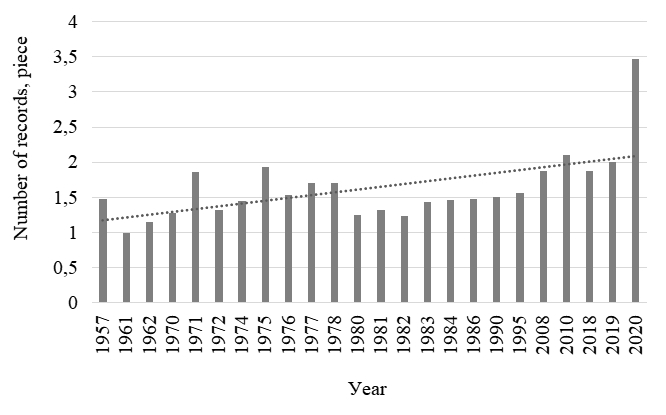
Number of records in different observation periods.

The first step was to collate a general list of zooplankton species (including rotifers and crustaceans) found in the Middle Volga River Basin. All records are confirmed by collection samples stored at the Institute of Ecology of the Volga Basin, Russian Academy of Sciences. The earliest records on the species composition of zooplankton in the Middle Volga date back to 1957, the latest being obtained in 2020 (Fig. 3). The increase in the number of finds in 2020 is associated with an increase in interest in studying the species composition of zooplankton in various biotopes (littoral, pelagial), as well as ongoing monitoring by employees of the Institute of Ecology of the Volga Basin, Russian Academy of Sciences.

In addition, some species are expanding their areas and actively conquer new reservoirs. For example, in the first decade of the XXI century, the rotifer *Kellicottiabostoniensis* (Rousselet, 1908), an invader species of American origin, has been found more frequently in Russia than before. By 2015, *K.bostoniensis* was found in more than 40 different types of water bodies and watercourses of the European part of Russia. This rotifer species is widespread; it has become a common species in forest lakes and rivers of the Baltic Sea Basin and the Volga-Baltic watershed area. In the Volga River Basin, it settled south to 55° N (lakes of the Basins of the Oka and Pra Rivers) and to the east almost up to 45° E (Kerzhenets River, Basin of the Cheboksary Reservoir) (Zhdanova et al. 2016). We noted the first finds of *K.bostoniensis* in Usinsky Bay of the Kuibyshev Reservoir in autumn of 2020.

### Funding

The study was carried out within the framework of the State Program for Basic Research for 2013–2021, projects nos. АААА-F17-117112040039-7 and АААА-F17-117112040040-3

## Sampling methods

### Study extent

**Figure 4.**
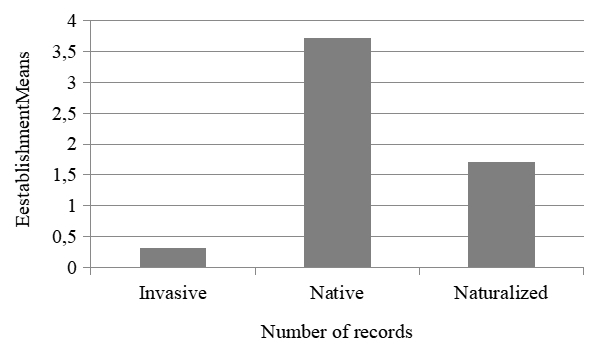
Number of records in the dataset characterising zooplankton in relation to its status.

The presented dataset on taxonomic compositions and abundance of zooplankton in the Middle Volga River Basin is based on published materials (Kutikova 1970, Mordukhay-Boltovskoy 1975, Timokhina 2000, Romanova et al. 2005, Popov 2006, Mukhortova 2008, Alekseev and Tsalolikhin 2010, Romanova 2010, Mukhortova and Sabitova 2014, Rubanova et al. 2020) and the authors' original materials (samples). The species list includes native species and naturalised species, including invasive species. The dataset contains mainly native species (98.5%), while naturalied (including invasive) species account for no more than 1.5% (Fig. 4). An invasive species is an alien species that actively displaces native species. (Zhdanova et al. 2016, Lazareva 2020, Shurganova et al. 2021).

### Sampling description

The identification of the species composition of zooplankton was performed in 1957–2020. Standard hydrobiological methods for studying zooplankton were applied (Mordukhay-Boltovskoy 1975, Alekseev and Tsalolikhin 2010). Sampling grids have been developed. Monographs (e.g. Timokhina 2000, Popov and Mukhortova 2016) and review articles (e.g. Timokhina 1983, Mukhortova 2010, Popov 2013, Mukhortova and Sabitova 2021) were used to compile the list of species.

### Quality control

All samples were identified by the researchers working in the Institute of Ecology of the Volga Basin, Russian Academy of Sciences and were stored in the scientific collection of the Institute. The reliability of the taxonomic definitions was confirmed by taxonomists of A.N. Severtsov Institute of Ecology and Evolution, Russian Academy of Sciences (Sinev and Gavrilko 2020, Sinev et al. 2020, Korovchinsky et al. 2021). The taxonomic nomenclature is given in accordance with the taxonomic system GBIF Backbone Taxonomy (GBIF Secretariat 2021). In order to publish the dataset on the GBIF network, the records have been adjusted according to the Darwin Core specifications (Wieczorek et al. 2012).

### Step description

**Figure 5.**
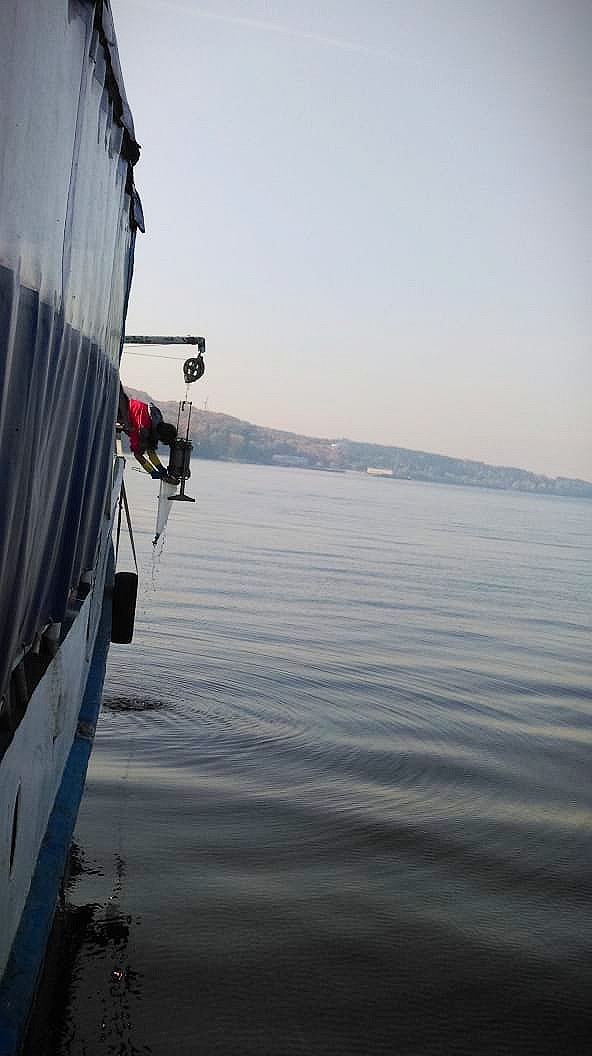
Monitoring of the water bodies: sampling of zooplankton in the pelagic zone of the Priplotinny Ples Reach of the Kuibyshev Reservoir (by means of a Dyachenko bathometer).

**Figure 6.**
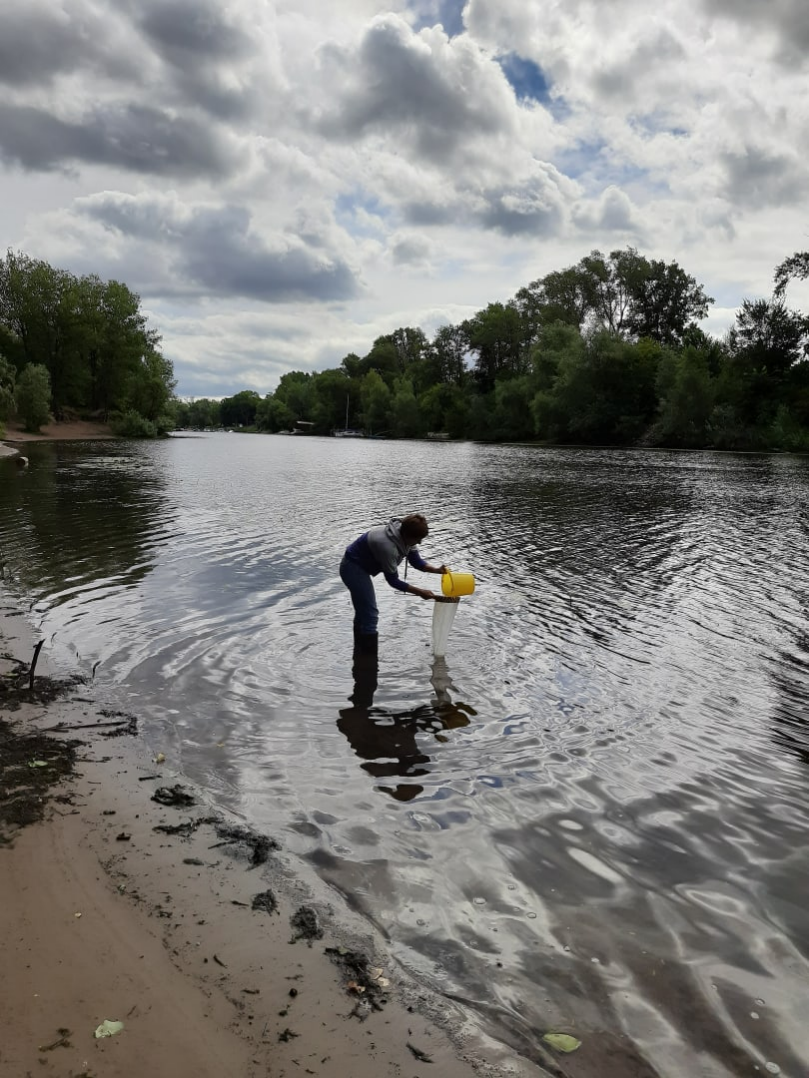
Monitoring of the water bodies: sampling of zooplankton in the littoral (water filtering through an Apstein plankton net, mesh size of 99 μm).

1. The materials presented in scientific monographs served as the initial data for developing a complete list of zooplankton species in the Middle Volga River Basin (Timokhina 2000, Chuikov 2000, Popov and Mukhortova 2016).

2. The obtained information is supplemented by the results of field sampling of zooplankton, which have been carried out regularly in the study area (Figs 5, 6). In the Kuibyshev Reservoir, 10 dm^3^ of water were taken from standard depths (0–32-m water column) by a Dyachenko bathometer; the water samples were sieved for zooplankton through a plankton net with a 99-µm mesh, equipped with a 0.1-dm^3^ cod end.

From 1957—2006, the zooplankton sampling was also performed with a 10-dm^3^ Dzyuban bathometer and a Juday net (nylon sieve, 99-µm mesh, 0.1-dm^3^ cod end). In the Saratov Reservoir and lakes, zooplankton samples were taken also with a 4-dm^3^ Ruthner bathometer and concentrated through a plankton net as described above (Mordukhay-Boltovskoy 1975).

The percentage of records in the dataset of the samples collected with the Dyachenko bathometer is 92% of total number of samples, Dzyuban bathometer, 1%, Ruthner bathometer, 0.5% and Juday net, 7%.

3. The zooplankton species were identified using the taxonomic keys for local fauna (Kutikova 1970, Alekseev and Tsalolikhin 2010).

4. The calculation of the zooplankton occurrence (ind.) was carried out according to an accepted standard (Bolotov 2012).

5. The dataset field names were chosen according to the Darwin Core (Wieczorek et al. 2012) and include the following: «eventID», «occurrenceID», «scientificName», «taxonRank», «kingdom», «family», «genus», «specificEpithet», «infraspecificEpithet», «establishmentMeans», «samplingProtocol», «sampleSizeValue», «sampleSizeUnit», «individualCount», «eventDate», «basisOfRecord», «occurrenceStatus», «recordedBy», «identifiedBy», «higherGeographyID», «country», «countryCode», «stateProvince», «county», «waterBody», «decimalLatitude», «decimalLongitude», «coordinateUncertaintyInMetres», «geodeticDatum», «minimumDepthInMetres», «maximumDepthInMetres», «verbatimDepth», «georeferencedBy», «institutionCode», «language», «associatedReferences».

## Geographic coverage

### Description

The Volga River is located in the European part of Russia. It is one of the largest rivers worldwide, the world's largest river flowing into a closed body of water and the largest river in terms of water content, watershed area and length in Europe. The studies were carried out in the Middle Volga River Basin, namely, in the Kuibyshev Reservoir (Samara Oblast and the Republic of Tatarstan) and Saratov Reservoir (Samara Oblast), in several lakes (Raifskoe, Gniloe, Krugloe and Lenevo), located in the Volzhsko-Kamsky State Biosphere Reserve (Republic of Tatarstan) and in Lake Aslikul, one of the largest lakes of the Middle Volga River Basin, located in the Asly-Kul Natural Park (Republic of Bashkortostan).

**Figure 7.**
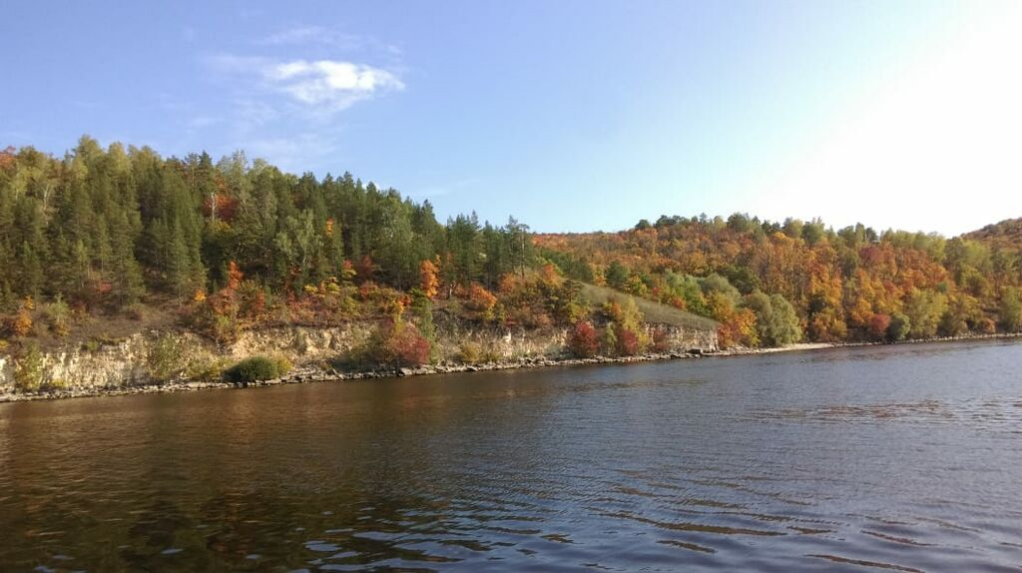
Zooplankton sampling sites: littoral of the Kuibyshev Reservoir.

**Figure 8.**
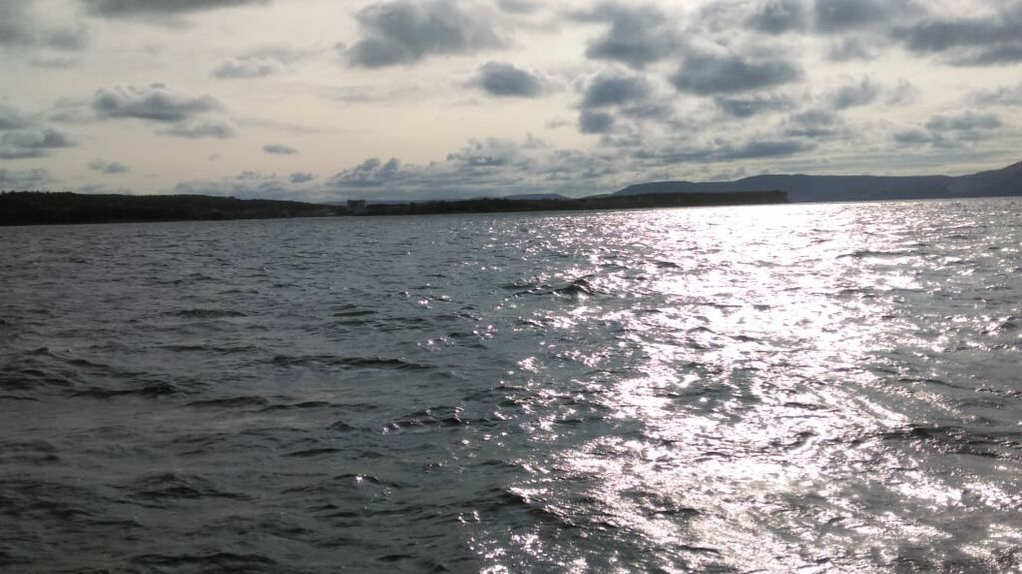
Zooplankton sampling sites: pelagial of the Kuibyshev Reservoir.

**Figure 9.**
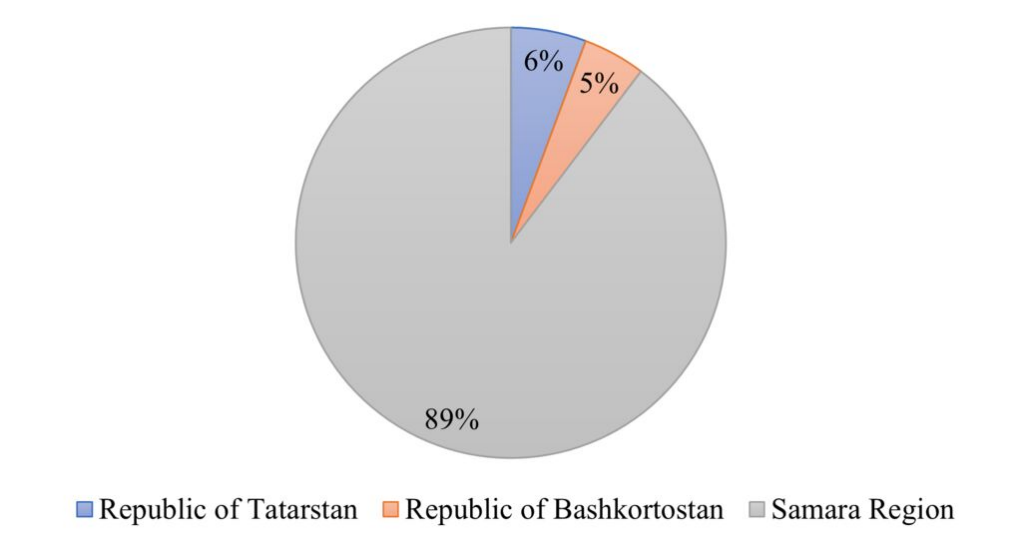
Distribution of the number of records by regions of the Russian Federation.

1. The length of the Kuibyshev Reservoir is 510 km, the greatest width is 40–44 km at the mouth of the Kama River, the water surface area is 6,450 km² (the second largest riverine reservoir in the world); 50.7% of the area is located in the Republic of Tatarstan); the total water volume is 58 km³, useful volume is 34 km³. The backwater level at the dam of the Zhigulevskaya HPP is 29 m (Figs 7, 8).
2. The length of the Saratov Reservoir is 341 km, the maximum width is 0.8–12 km, the water area at a normal retaining level is 1,831 km², the total volume is 12.9 km³ and the depth is 8–28 m. The flow rate is 0.27–0.56 m/sec.
3. Volzhsko-Kamsky State Biosphere Reserve was established in 1960; since 2005, it has been included in the UNESCO system of biosphere reserves. It is located in the Republic of Tatarstan, consisting of two sites: Raifsky (5,921 hectares, Zelenodolsky District) and Saralinsky (4,170 hectares, Laishevsky District). The water area of the reserve covers is 1,300 hectares.
4. Lake Aslikul is the largest lake in the Middle Volga River Basin, located in the Asly-Kul Natural Park (Republic of Bashkortostan). The water surface area is 22–23.5 km², the lake volume is 0.119 km³, the average depth is 5.1 m. It has a karst-hole origin.
5. Administratively, the study area belongs to the Samara Oblast, the Republic of Tatarstan and Republic of Bashkortostan, the Russian Federation (Fig. 9).

### Coordinates

53.149920 and 55.917733 Latitude; 48.612276 and 54.623405 Longitude.

## Taxonomic coverage

### Description

The dataset includes the records on the taxonomic diversity of zooplankton in the Middle Volga River Basin, represented by 111 species (including 17 subspecies), 45 genera, 25 families, one superorder and two phyla (Table 1).

**Figure 10.**
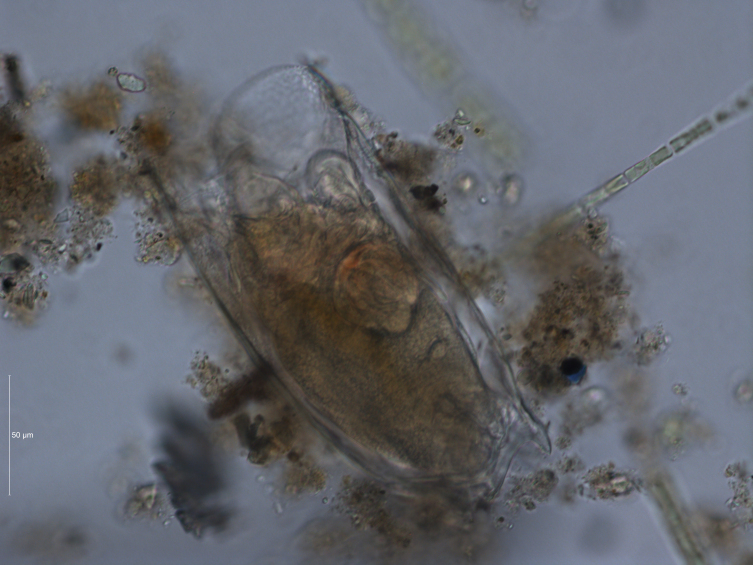
*Mytilinaventralisventralis* (Ehrenberg, 1832), total preparations, magnification 400×, Leica DM5500 B: dorsal view. Photo by S.V. Bykova (Institute of Ecology of the Volga Basin, Russian Academy of Sciences).

**Figure 11.**
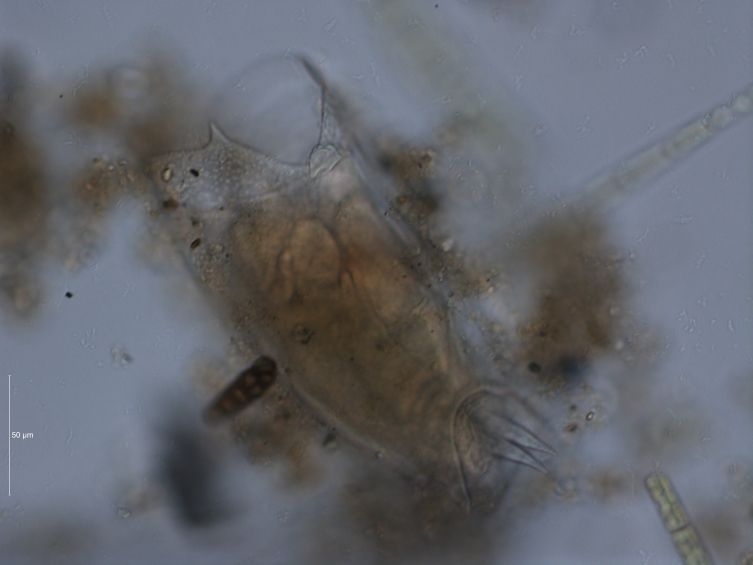
*Mytilinaventralisventralis* (Ehrenberg, 1832), total preparations, magnification 400×, Leica DM5500 B: side view. Photo by S.V. Bykova (Institute of Ecology of the Volga Basin, Russian Academy of Sciences).

One of the most common and abundant species of zooplankton is *Mytilinaventralisventralis*; it is a characteristic species of the Kuibyshev Reservoir (Figs 10, 11).

**Figure 12.**
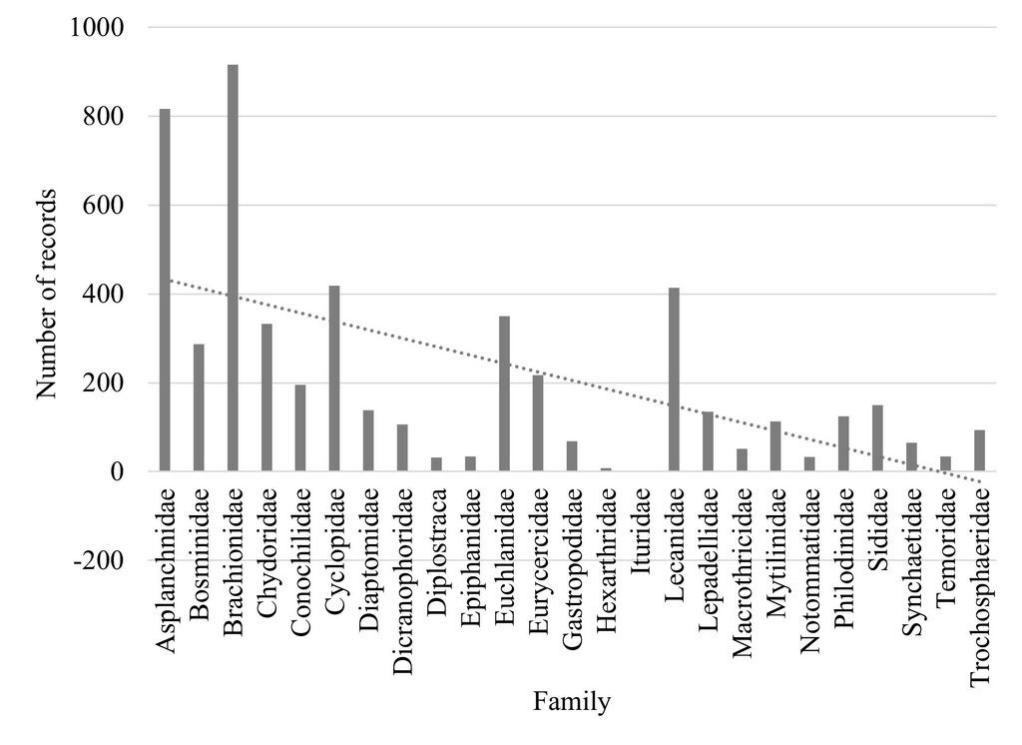
Distribution of dataset records by zooplankton families.

Families Asplanchnidae, Bosminidae, Brachionidae, Chydoridae, Conochilidae, Cyclopidae, Diaptomidae, Dicranophoridae, Diplostraca, Epiphanidae, Euchlanidae, Eurycercidae, Gastropodidae, Lecanidae, Lepadellidae, Macrothricidae, Mytilinidae, Philodinidae, Sididae, Synchaetidae and Trochosphaeridae are the most diverse, accounting for 80% of records (Fig. 12).

Several families of Rotifera are represented by a single species (~ 1% of records): Hexarthridae (*Hexarthramira* (Hudson, 1871)), Animalia (*Ituraaurita* (Ehrenberg, 1838)) and Notommatidae (*Eosphoranajas* Ehrenberg, 1830). Temoridae family (*Eurytemoravelox* (Lilljeborg, 1853)), which accounts for 0.5% of the records, are rare representatives of Crustacea (Fig. 12).

The occurrence of zooplankton in the samples collected varied from 1 ind. to 3161 ind. (on average ~ 87.01 ± 123 ind.).

The zooplankton abundance in different water bodies ranged from 0.33 to 1,046 thousand ind./m^3^ (on average ~ 13.79 ± 50.51 thousand ind./m^3^).

## Temporal coverage

### Notes

**Figure 13.**
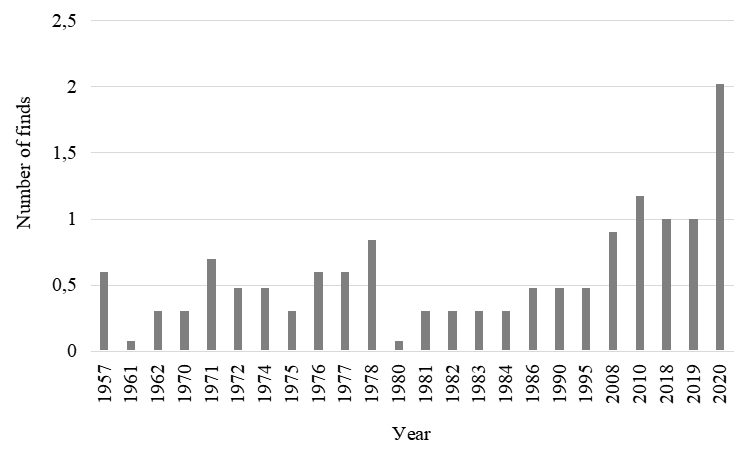
Number of findings (species) in different observation years.

The presented dataset contains information on the occurrence of zooplankton species before the regulation of reservoirs since 1957, while the most recent observations are made in 2020 (Fig. 13).

## Collection data

### Collection name

The zooplankton collections of the Institute of Ecology of the Volga Basin of the Russian Academy of Sciences

### Specimen preservation method

wet

## Usage licence

### Usage licence

Creative Commons Public Domain Waiver (CC-Zero)

### IP rights notes

This work is licensed under a Creative Commons Attribution (CC-BY) 4.0 License.

## Data resources

### Data package title

Distribution and species composition of zooplankton (rotifers and crustaceans) in the Basin of the Middle Volga River, Russia

### Resource link

https://doi.org/10.15468/k96rq7

### Alternative identifiers

https://www.gbif.org/dataset/cc76b68b-87cc-442b-9a59-faef85741cc4

### Number of data sets

1

### Data set 1.

#### Data set name

Information on the distribution, occurrence and abundance of zooplankton in the Basin of the Middle Volga River, Russia

#### Data format

Darwin Core

#### Number of columns

36

#### Download URL

https://www.gbif.org/dataset/cc76b68b-87cc-442b-9a59-faef85741cc4

#### Description

The presented dataset contains information on the distribution and species composition of zooplankton (rotifers and crustaceans) registered in the Basin of the Middle Volga River, Russia. The hydrobiological data were obtained and published from 1957 to 2020. In total, the dataset includes 5141 records of 111 zooplankton species (including 17 subspecies), belonging to 45 genera.

**Data set 1.** 

Column label	Column description
eventID	An identifier for the set of information associated with an Event (something that occurs at a place and time). May be a global unique identifier or an identifier specific to the dataset.
occurrenceID	An identifier for the Occurrence (as opposed to a particular digital record of the occurrence). In the absence of a persistent global unique identifier, construct one from a combination of identifiers in the record that will most closely make the occurrenceID globally unique.
scientificName	The full scientific name, with authorship and date information, if known. When forming part of an Identification, this should be the name in the lowest level taxonomic rank that can be determined. This term should not contain identification qualifications, which should instead be supplied in the IdentificationQualifier term.
taxonRank	The taxonomic rank of the most specific name in the scientificName.
kingdom	The full scientific name of the kingdom in which the taxon is classified.
family	The full scientific name of the family in which the taxon is classified.
genus	The full scientific name of the genus in which the taxon is classified.
specificEpithet	The name of the first or species epithet of the scientificName.
infraspecificEpithet	The name of the lowest or terminal infraspecific epithet of the scientificName, excluding any rank designation.
establishmentMeans	The process by which the biological individual(s) represented in the Occurrence became established at the location.
samplingProtocol	The methods or protocols used during an Event, denoted by an IRI.
sampleSizeValue	A numeric value for a measurement of the size (time duration, length, area or volume) of a sample in a sampling event.
sampleSizeUnit	The unit of measurement of the size (time duration, length, area or volume) of a sample in a sampling event.
individualCount	The number of individuals present at the time of the Occurrence.
eventDate	The date-time or interval during which an Event occurred. For occurrences, this is the date-time when the event was recorded. Not suitable for a time in a geological context.
basisOfRecord	The specific nature of the data record.
occurrenceStatus	A statement about the presence or absence of a Taxon at a Location.
recordedBy	A list (concatenated and separated) of names of people, groups or organisations responsible for recording the original Occurrence. The primary collector or observer, especially one who applies a personal identifier (recordNumber), should be listed first.
identifiedBy	A list (concatenated and separated) of names of people, groups or organisations who assigned the Taxon to the subject.
higherGeographyID	An identifier for the geographic region within which the Location occurred.
country	The name of the country or major administrative unit in which the Location occurs.
countryCode	The standard code for the country in which the Location occurs.
stateProvince	The name of the next smaller administrative region than country (state, province, canton, department, region etc.) in which the Location occurs.
county	The full, unabbreviated name of the next smaller administrative region than stateProvince (county, shire, department etc.) in which the Location occurs.
waterBody	The name of the water body in which the Location occurs.
decimalLatitude	The geographic latitude (in decimal degrees, using the spatial reference system given in geodeticDatum) of the geographic centre of a Location. Positive values are north of the Equator, negative values are south of it. Legal values lie between -90 and 90, inclusive.
decimalLongitude	The geographic longitude (in decimal degrees, using the spatial reference system given in geodeticDatum) of the geographic centre of a Location. Positive values are east of the Greenwich Meridian, negative values are west of it. Legal values lie between -180 and 180, inclusive.
coordinateUncertaintyInMetres	The horizontal distance (in metres) from the given decimalLatitude and decimalLongitude describing the smallest circle containing the whole of the Location. Leave the value empty if the uncertainty is unknown, cannot be estimated or is not applicable (because there are no coordinates). Zero is not a valid value for this term.
geodeticDatum	The ellipsoid, geodetic datum or spatial reference system (SRS) upon which the geographic coordinates given in decimalLatitude and decimalLongitude are based.
minimumDepthInMetres	The lesser depth of a range of depth below the local surface, in metres.
maximumDepthInMetres	The greater depth of a range of depth below the local surface, in metres.
verbatimDepth	The original description of the depth below the local surface.
georeferencedBy	A list (concatenated and separated) of names of people, groups or organisations who determined the georeference (spatial representation) for the Location.
institutionCode	The name (or acronym) in use by the institution having custody of the object(s) or information referred to in the record.
language	The language of the resource
associatedReferences	A list (concatenated and separated) of identifiers (publication, bibliographic reference, global unique identifier, URI) of literature associated with the Occurrence.

## Figures and Tables

**Table 1.** General taxonomic ranking of zooplankton species.

Phylum, Order	Absolute number			Records
	Families	Genera	Species	
Rotifera	17	24	69	3477
Arthropoda	8	21	42	1664
including Crustacea:				
Cyclopiformes	1	4	10	409
Calaniformes	2	4	5	171
Total	25	45	111	514
